# High-Sensitivity C-Reactive Protein Elevation Is Independently Associated with Subclinical Renal Impairment in the Middle-Aged and Elderly Population—A Community-Based Study in Northern Taiwan

**DOI:** 10.3390/ijerph17165878

**Published:** 2020-08-13

**Authors:** Hai-Hua Chuang, Rong-Ho Lin, Wen-Cheng Li, Wei-Chung Yeh, Yen-An Lin, Jau-Yuan Chen

**Affiliations:** 1 Department of Family Medicine, Chang-Gung Memorial Hospital, Taipei & Linkou Branch, Taoyuan 33305, Taiwan; chhaihua@gmail.com (H.-H.C.); 620313@cgmh.org.tw (W.-C.L.); sendoh777777@gmail.com (W.-C.Y.); s19401044@gmail.com (Y.-A.L.); 2 College of Medicine, Chang Gung University, Taoyuan 33302, Taiwan; 3 Department of Industrial Engineering and Management, National Taipei University of Technology, Taipei 10608, Taiwan; rhlin@mail.ntut.edu.tw; 4 Obesity Institute & Genomic Medicine Institute, Geisinger, Danville, PA 17822, USA

**Keywords:** cardiometabolic risk factors, chronic kidney disease, community medicine, high-sensitivity C-reactive protein, renal impairment

## Abstract

This cross-sectional study aimed to investigate the associations between high-sensitivity C-reactive protein (hs-CRP) and renal impairment (RI) among middle-aged and elderly people. We collected and analyzed demographic, anthropometric, metabolic, and renal function data in a community-based population in Northern Taiwan. We excluded subjects with acute inflammation from this study and defined RI as the presence of urinary albumin–creatinine ratio 30–300 mg/g or an estimated glomerular filtration rate of <60 mL/min/1.73 m^2^. There were 131, 125, and 125 participants in the low (≤0.80 mg/L), middle (0.81–1.76 mg/L), and high (>1.77 mg/L) hs-CRP tertiles, respectively. hs-CRP exhibited significantly positive correlations with body mass index, waist circumference, systolic blood pressure, triglyceride, and fasting plasma glucose, and a negative correlation with high-density lipoprotein. The prevalence and odds ratio of RI significantly increased across hs-CRP tertiles from low to high, and this trend remained significant after adjusting for the conventional cardiometabolic risk factors. hs-CRP ≥ 1.61 mg/L in the total group and ≥2.03 mg/L in the elderly group accurately predicted RI (*p* = 0.01 and 0.03, respectively). These findings suggest that we should carefully evaluate the renal function for at-risk individuals with hs-CRP elevation.

## 1. Introduction

High-sensitivity C-reactive protein (hs-CRP) is considered to be an indicator of systemic inflammation. hs-CRP is also elevated in patients with cardiometabolic risk factors such as metabolic syndrome (MetS) and its components, including obesity, elevated blood pressure, dyslipidemia, and hyperglycemia [1,2].

Renal impairment (RI) is one of the major complications in patients with the abovementioned cardiometabolic risk factors [3]. As the disease progresses, mild and subclinical RI eventually develop into chronic kidney disease (CKD) or even end-stage renal disease (ESRD), which leads to a compromised quality of life and shortened life expectancy of the patients, consequently resulting in a heavy socioeconomic burden. CKD is an increasingly prevalent condition that is estimated to affect 10–12% of the global population, and is recognized as a public health priority [4]. While the prevalence of ESRD in Taiwan is the highest in the world, the prevalence of CKD is also considerably high: 11.93% among adults of all ages (≥20 years old) and 37.2% in the elderly population (≥65 years old). Early intervention of CKD results in better health outcomes and lower medical costs. Unfortunately, most patients are not diagnosed and treated until they reach later stages of the disease.

The pathophysiology of renal function decline in patients with cardiometabolic risk factors is multifactorial, and one of the proposed mechanisms is through systemic inflammation associated with insulin resistance [5,6]. As a surrogate marker of systemic inflammation, hs-CRP is suggested to be linked to CKD in the literature [7,8]. However, the relationship between hs-CRP and subclinical RI in the middle-aged and elderly population in Taiwan is not yet well established.

We hypothesized that, in addition to being positively associated with cardiometabolic risk factors, hs-CRP was positively and independently associated with subclinical RI. The aim of the present study was to assess the associations between hs-CRP, cardiometabolic risk factors, and subclinical RI in a middle-aged and elderly population in Taiwan.

## 2. Materials and Methods

### 2.1. Study Design and Subjects

**Figure 1:**
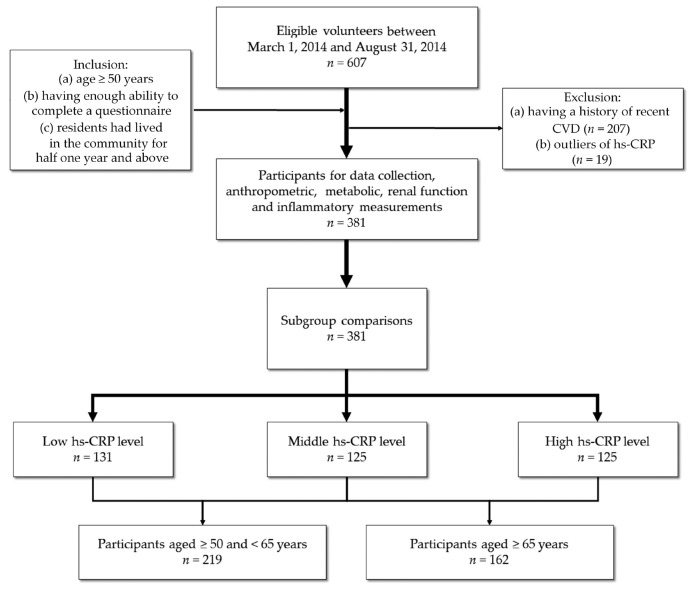
Study flow chart. Initially, we recruited 607 eligible volunteers to participate in a community health promotion project; however, we excluded 11 cases with a history of recent cardiovascular disease (CVD), 19 outliers of high-sensitivity C-reactive protein (hs-CRP) (>10 mg/L), and 196 that declined to participate; therefore, we statistically analyzed 381 participants in the present study.

This was a cross-sectional quantitative study. Data for this study were collected from a community-based health promotion project “Health Screening in the Middle-elderly and Health Promotion Effectiveness of Intervention in Guishan Township, Taoyuan County”. The project was conducted from March to August 2014 by a medical center in northern Taiwan to its nearby communities. The project was approved by Chang Gung Medical Foundation Institutional Review Board on 2013/08/16 (102-2304B). Residents older than 50 years of age were recruited and completed a questionnaire during a face-to-face interview. Each subject was enrolled voluntarily and provided written informed consent [1]. The inclusion criteria were: (a) age ≥50 years; (b) having enough ability to complete a questionnaire; and (c) residents had lived in the community for ≥6 months. The exclusion criteria were: (a) having a history of recent cardiovascular disease (CVD); (b) outliers of hs-CRP; and (c) declining to participate (Figure 1).

### 2.2. Data Collection and Parameter Measurements

Data collection included background information, medical history, anthropometric measurements, metabolic profiles, renal function studies, and inflammatory markers. Background information included age, sex, alcohol consumption, smoking status, marital status, Chinese herb use, nonsteroidal anti-inflammatory drug (NSAID) use, hypertension, diabetes mellitus, and hyperlipidemia. Anthropometric measurements included body height (BH), body weight (BW), and waist circumference (WC). Body mass index (BMI) was calculated as the ratio between BW and BH in meters squared (kg/m^2^). Metabolic profiles included blood pressure (BP), lipid profile (total cholesterol; TC), low-density lipoprotein (LDL), high-density lipoprotein (HDL), triglycerides (TGs), and fasting plasma glucose (FPG). The renal function study measured the serum creatinine and urine albumin/creatinine ratio (ACR). The inflammatory marker was hs-CRP. To exclude acute inflammation from this study, we further excluded subjected with hs-CRP > 10 mg/L [9].

### 2.3. Definition of Renal Impairment (RI)

The estimated glomerular filtration rate (eGFR) was calculated using a modified version of the Modification of Diet in Renal Disease equation for Chinese CKD patients: 175 × (creatinine)^−1.234^ × (age)^−0.179^ × 0.79 (for females). RI was defined as the presence of kidney damage (urine ACR ≥ 30 mg/g and <300 mg/g) or decreased renal function with eGFR < 60 mL/min/1.73 m^2^. We chose subclinical RI as the term for our main outcome of interest instead of CKD, since this was a cross-sectional study, and it might not have been correct to make a diagnosis of CKD based on a one-time urine and blood test from the subjects.

### 2.4. Statistical Analysis

Statistical analysis was conducted using SPSS Statistics Version 22 (IBM, SPSS, Armonk, NY, USA). A *p*-value < 0.05 was considered statistically significant. Most of the distributions of the variables were non-normal, assessed using the Kolmogorov–Smirnov test.

Subjects were stratified into tertiles according to hs-CRP levels. Clinical characteristics were compared among tertiles using one-way analysis of variance (one-way ANOVA) for continuous variables and chi-square test for categorical variables. The correlation between hs-CRP and cardiometabolic risk factors was examined with Spearman’s correlation test. The association between tertiles of hs-CRP and RI was analyzed using multiple logistic regression; model 1 was unadjusted; model 2 was adjusted for age and sex; and model 3 was adjusted for age, sex, smoking, BMI, systolic BP, LDL, FPG, Chinese herb use, and NSAID use. The Cochran–Armitage trend test was used to evaluate the increasing prevalence of RI as a function of hs-CRP level tertiles. Areas under the receiver operating characteristic curve (AUCs) were used to examine the ability of hs-CRP to predict RI, and the optimized cutoff points for hs-CRP, sensitivity, and specificity were acquired using the maximal Youden Index.

## 3. Results

### 3.1. General Characteristics of the Study Population According to Tertiles of hs-CRP Levels

There were 607 eligible volunteers who attended the community screening, among whom 11, 19, and 196 were excluded due to recent CVD, extreme values of hs-CRP, and declining to participate, respectively. A final total of 381 subjects, including 134 men and 247 women, with a mean age of 64.55 ± 8.48 years, were enrolled for the analysis. None of the subjects reported symptoms of advanced CKD. Subjects were categorized into tertiles based on their levels of hs-CRP. There were 131, 125, and 125 subjects in the low, middle, and high tertiles, respectively. BMI, WC, SBP, hs-CRP, HDL, LDL, TGs, urinary ACR, and RI were significantly different across the tertiles. There was no significant difference observed for other variables (Table 1).

**Figure 2:**
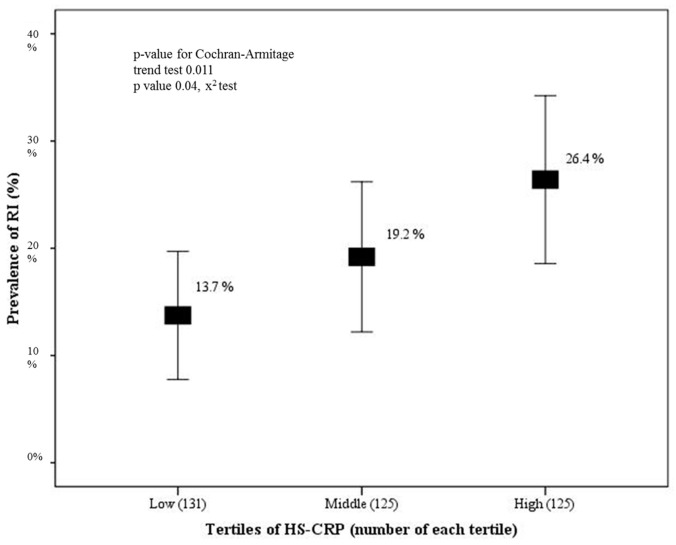
Prevalence of RI according to tertiles of hs-CRP levels. There was a modest linearly increasing trend across hs-CRP tertiles.

Figure 2 demonstrates the prevalence of RI according to tertiles of hs-CRP levels. The prevalence was 13.7%, 19.2%, and 26.4% in the low, middle, and high tertiles, respectively. The trend test and chi-square (χ^2^) test were significant.

### 3.2. Correlations Between hs-CRP and Cardiometabolic Risk Factors

hs-CRP had a significant positive correlation with BMI, WC, SBP, FPG, LDL, and TGs. In contrast, hs-CRP and HDL were inversely correlated. After adjusting for age, the aforementioned findings remained significant (Table 2).

### 3.3. Associations between Tertiles of hs-CRP and RI

The odds ratio (OR) of RI significantly increased in the high tertile using the low tertile as a reference. This finding was significant, with an OR of 2.25 in model 1 (unadjusted), an OR of 2.22 in model 2 (adjusted for age and sex), and an OR of 2.16 in model 3 (adjusted for age, sex, smoking, BMI, SBP, LDL, FPG, Chinese herb use, and NSAID use). Across increasing hs-CRP tertiles, the trend test of increasing RI was also significant in all three models (Table 3).

**Figure 3:**
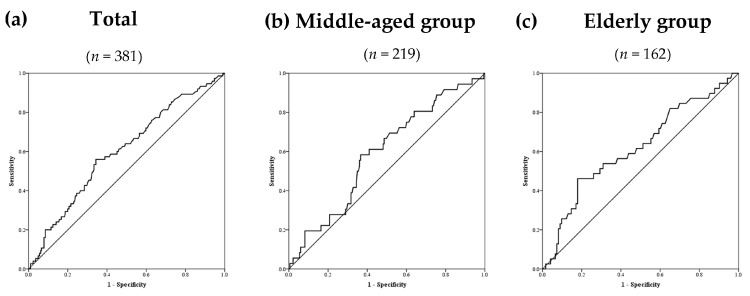
The receiver operating characteristic curves for high-sensitivity C-reactive protein as a predictor of renal impairment in the total group (**a**), middle-aged group (**b**), and elderly group (**c**).

Figure 3 demonstrates the receiver operating characteristic curves for hs-CRP to predict RI in the total (Figure 3a), middle-aged (Figure 3b), and elderly (Figure 3c) groups. Table 4 summarizes the areas under the receiver operating characteristic curve (AUCs), sensitivities, and specificities according to the optimized cutoff points of hs-CRP for predicting RI in these three groups. Based on a cutoff point of 1.61 mg/dL, hs-CRP accurately predicted RI in the total group (AUC = 0.60; *p* = 0.01; sensitivity = 0.56; specificity = 0.66). Although the predictive value of hs-CRP (cutoff point = 2.03 mg/dL) in the elderly group was promising (AUC = 0.62; *p* = 0.03; sensitivity = 0.46; specificity = 0.82), its predive value (cutoff point = 1.61 mg/dL) was not significant in the middle-aged group (AUC = 0.59; *p* = 0.09; sensitivity = 0.58; specificity = 0.63).

## 4. Discussion

In our study, hs-CRP was positively associated with conventional cardiometabolic risk factors including BMI, WC, SBP, FPG, LDL, and TGs were negatively associated with HDL. Furthermore, when we divided subjects into tertiles based on hs-CRP levels, the aforementioned variables were significantly different across each group. These findings are compatible with previous reports showing that the level of hs-CRP is highly related to risk factors of cardiovascular diseases (CVD) [10,11,12]. The results of this study validate our hypothesis; the prevalence of subclinical RI increased across the tertiles of hs-CRP from low to high, and the trend remained significant even after adjusting for many traditional risk factors. Obesity, hypertension, dyslipidemia, and diabetes are well-recognized, major risk factors for kidney damage [13,14]. Diabetic nephropathy is the leading cause of renal failure in many developed countries. However, our study further shows that even after adjusting for conventional risk factors, Chinese herb use, and NSAID use, hs-CRP was independently related to subclinical RI. Notably, hs-CRP was more strongly related to elevated ACR than decreased eGFR and predicted subclinical RI in the elderly population more effectively than in the middle-aged population.

To address the relationship between hs-CRP and subclinical RI, we discuss it from three distinct viewpoints: (1) the biology of hs-CRP as a biomarker of inflammation; (2) the role of chronic inflammation in RI; and (3) the possibility of using hs-CRP in risk stratification for RI. The outcome in this study was RI; however, since CKD is the expected complication when RI lasts over time, we mention both terms frequently as appropriate in the following discussion.

### 4.1. Biology of hs-CRP as a Biomarker of Inflammation

CRP, a member of the pentraxin family, is a ring-shaped protein found in blood plasma that is mainly secreted and released by hepatocytes. CRP is measured as hs-CRP in low-level inflammatory conditions. The production of hs-CRP is triggered by several cytokines, such as interleukin-6 (IL-6). IL-6 is released from activated leukocytes in response to infection or trauma and from vascular smooth muscle cells in response to atherosclerosis [12,15].

hs-CRP is a circulating reactant associated with a wide range of acute and chronic inflammatory conditions. When there is an acute stimulus such as viral or bacterial infection, the level of hs-CRP can increase as much as 100 to 500 mg/L within 4 to 6 h, doubling every 8 h, and can increase up to 10,000-fold in severe cases. The increase in hs-CRP levels tends to be proportional to the intensity of inflammation. hs-CRP levels decrease quickly with a short half-life of approximately 4 to 7 h when inflammation subsides. The above characteristics make hs-CRP a particularly useful clinical biomarker to reflect the presence and degree of inflammation [15,16]. hs-CRP concentrations between 2 and 10 mg/L are considered to indicate metabolic inflammation, which has been postulated as an important pathway linked to atherosclerosis and subsequent CVD. Abundant studies have reported a positive association between hs-CRP level and cardiometabolic risk factors [10,11].

### 4.2. Role of Chronic Inflammation in Subclinical RI

Under normal conditions, inflammation is a protective physiological response to harmful stimuli. However, in conditions such as chronic RI, when inflammation becomes persistent and deregulated, it becomes maladaptive and debilitating [17]. The role of systemic inflammation in chronic RI has been prodigiously investigated, especially in the context of ESRD [18]. Systemic inflammation is considered to be not only a risk factor for mortality but also a catalyst for other complications, and is related to a premature aging phenotype. The pathophysiological mechanisms underlying this process are complicated and intricate, such as premature aging of the immune system, defective regulation of inflammatory processes, abnormalities in mineral metabolism, and gut dysbiosis [19].

The other factor that inflammation and RI are both linked to is atherosclerosis. The concept of inflammation being central to the initiation and progression of atherosclerotic changes is now mature, originating from observations in the 1800s. Atherosclerosis is now considered an inflammatory disease characterized by the progressive accumulation of lipids in the vessel wall [20,21]. Atherosclerosis leads to CVDs such as coronary artery disease, stroke, and peripheral vascular disease [22]. RI, as a common comorbidity among patients with CVD, has been postulated as both a predisposing factor and a consequence of atherosclerosis. RI accelerates atherosclerosis via the augmentation of inflammation, perturbation of lipid metabolism, and other mechanisms [19], which in turn can contribute to the progression of renal decline [13,23].

### 4.3. Possibility of Using hs-CRP in Risk Stratification for RI

Patients with CKD have a considerably higher incidence of CVD events and premature death. A substantial volume of research has focused on finding biomarkers for better risk stratification in this population. hs-CRP is one of the earliest and most frequently used biomarkers. The positive association between hs-CRP level and CVD events, as well as overall morality in CKD patients, is evident [24,25,26], although some still debate its clinical application [27]. Some other parameters have also been suggested to serve this purpose, such as microalbuminuria, natriuretic peptides, troponins, adiponectin, leptin, phosphorus, parathyroid hormone, vitamin D, fibroblast growth factor 23, and matrix metalloproteinases [27,28].

While the literature extensively addresses hs-CRP for the prediction of outcomes of late-stage CKD patients, there is still limited research on the associations between hs-CRP and mild or early RI. A few studies have reported an inverse association between the hs-CRP level and GFR [29,30]. A recent study has also reported a link between hs-CRP elevation and urinary alpha-1 microglobulin (A1MG), an early sign of renal damage, in type II diabetes patients [31]. Our study provided evidence of the linkage of systemic inflammation and subclinical RI in seemingly healthy middle-aged and elderly community populations. This finding can help clinicians identify patients in need of more aggressive and focused preventive measures to improve future outcomes. Concerning the high prevalence of metabolic syndrome, as well as the high availability and easy interpretation of hs-CRP, this finding has practical clinical application.

The advantages of this study included a clear design, a sufficient sample size, a comprehensive inclusion of relevant confounders, and a well-performed data analysis. The novelty of this study was, from a community approach, to report different cutoff values of hs-CRP among the middle-aged and elderly cohorts to better predict subclinical renal impairment in these two specific age groups. However, the participants in our study were recruited from a few communities in northern Taiwan, and the characteristics of this cohort might differ from those of the general population. A possible selection bias and the single Han ethnicity of the subjects might limit the generalizability of this research. Also, RI was defined using a single urine and blood measurement, based on which the duration and nature of RI were not easy to clarify. Additionally, the study was cross-sectional and thus not able to examine causal relationships. A prospective case–control study is warranted to externally verify our present findings.

## 5. Conclusions

hs-CRP is significantly and independently associated with subclinical RI. Careful evaluation and early intervention should be considered in the middle-aged and elderly population with systemic inflammation and other comorbidities. Future studies with a prospective design will be of interest.

## Figures and Tables

**Table 1:** General characteristics of the study population according to tertiles of hs-CRP levels.

hs-CRP Levels (mg/dL)					
Variables	Total	Low	Middle	High	*p*-Value
		(≤0.80 mg/L)	(0.81–1.76 mg/L)	(>1.77 mg/L)	
	(*n* = 381)	(*n* = 131)	(*n* = 125)	(*n* = 125)	
Age (years)	64.55 ± 8.48	64.47 ± 7.56	64.18 ± 8.20	65.02 ± 9.63	0.73
Middle-aged people, *n* (%)	219 (57.5)	71 (54.2)	73 (58.4)	75 (60.0)	0.62
Men, *n* (%)	134 (35.2)	51 (38.9)	41 (32.8)	42 (33.6)	0.53
Marital status (single), *n* (%)	74 (19.4)	29 (22.1)	19 (15.2)	26 (20.8)	0.33
Current smoking, *n* (%)	39 (10.2)	11 (8.4)	12 (9.6)	16 (12.8)	0.49
BMI (kg/m^2^)	24.53 ± 3.52	23.45 ± 3.38	24.62 ± 3.11 ^a^	25.57 ± 3.75 ^a^	<0.001 ***
Waist circumference (cm)	85.04 ± 9.59	81.91 ± 8.98	85.39 ± 9.09 ^a^	87.98 ± 9.78 ^a^	<0.001 ***
SBP (mmHg)	129.60 ± 16.57	126.22 ± 16.20	131.08 ± 16.26	131.66 ± 16.85 ^a^	0.02 *
DBP (mmHg)	77.00 ± 11.44	76.26 ± 10.65	77.31 ± 11.13	77.46 ± 12.54	0.66
hs-CRP (mg/L)	1.76 ± 1.72	0.52 ± 0.17	1.25 ± 0.29 ^a^	3.57 ± 1.93 ^a,b^	<0.001 ***
HDL-C (mg/dL)	54.83 ± 13.85	57.92 ± 15.33	55.34 ± 13.25 ^a^	51.09 ± 11.92 ^a^	<0.001 ***
LDL-C (mg/dL)	118.94 ± 32.04	111.77 ± 28.80	121.75 ± 32.69 ^a^	123.64 ± 33.51 ^a^	0.01 *
TG (mg/dL)	121.60 ± 65.96	112.76 ± 65.70	116.11 ± 61.72	136.34 ± 68.32 ^a,b^	0.01 *
FPG (mg/dL)	95.94 ± 24.98	92.26 ± 15.02	97.97 ± 33.45	97.77 ± 23.13	0.11
Creatinine (mg/dL)	0.77 ± 0.34	0.75 ± 0.31	0.77 ± 0.36	0.79 ± 0.35	0.63
eGFR (mL/min/1.73 m^2^)	112.76 ± 32.90	115.44 ± 31.42	112.71 ± 32.58	110.00 ± 34.71	0.42
ACR ≥ 30 mg/g, *n* (%)	69 (18.1)	16 (12.2)	23 (18.4)	30 (24.0) ^a^	0.05
RI, *n* (%)	75 (19.7)	18 (13.7)	24 (19.2)	33 (26.4) ^a^	0.04 *
Chinese herb use, *n* (%)	31 (8.14)	14 (10.69)	7 (5.60)	10 (8.00)	0.33
NSAID use, *n* (%)	29 (7.61)	13 (9.92)	7 (5.60)	9 (7.20)	0.42
HTN, *n* (%)	192 (50.39)	62 (47.33)	59 (47.20)	71 (56.80)	0.22
DM, *n* (%)	75 (19.69)	18 (13.74)	25 (20.00)	32 (25.60)	0.06
Hyperlipidemia, *n* (%)	249 (65.35)	77 (58.78)	83 (66.40)	89 (71.20)	0.11

Clinical characteristics are expressed as mean ± SD for continuous variables and *n* (%) for categorical variables. The *p*-values were derived from one-way analysis of variance (one-way ANOVA) for continuous variables and chi-square test for categorical variables. ^a^ *p* < 0.05 versus low group; ^b^ *p* < 0.05 versus middle group in the Bonferroni post hoc comparisons; * *p* < 0.05; *** *p* < 0.001. Abbreviations: SBP, systolic blood pressure; DBP, diastolic blood pressure; BMI, body mass index; ALT, alanine aminotransferase; eGFR, estimated glomerular filtration rate; FPG, fasting plasma glucose; HDL-C, high-density lipoprotein cholesterol; hs-CRP, high-sensitivity C-reactive protein; LDL-C, low-density lipoprotein cholesterol; TG, triglyceride; ACR, albumin to creatinine ratio; RI, renal impairment; NSAID, nonsteroidal anti-inflammatory drug.

**Table 2:** Correlation between hs-CRP and cardiometabolic risk factors.

hs-CRP (*n* = 381)				
Variables	Unadjusted		Adjusted for Age	
	Spearman’s Coefficient	*p*-Value	Spearman ‘s Coefficient	*p*-Value
Age (years)	−0.02	0.74	NA	NA
BMI (kg/m^2^)	0.28	<0.001 ***	0.23	<0.001 ***
Waist circumference (cm)	0.30	<0.001 ***	0.23	<0.001 ***
SBP (mmHg)	0.18	<0.001 ***	0.12	0.03
DBP (mmHg)	0.06	0.22	0.06	0.26
FPG (mg/dL)	0.12	0.02 *	0.15	0.004
HDL-C (mg/dL)	−0.20	<0.001 ***	−0.16	0.002
LDL-C (mg/dL)	0.17	<0.001 ***	0.10	0.05
TG (mg/dL)	0.25	<0.001 ***	0.17	0.001
eGFR (mL/min/1.73 m^2^)	−0.06	0.24	−0.03	0.53
ACR (mg/g)	0.16	0.001 **	0.11	0.03 *

Abbreviations: SBP, systolic blood pressure; DBP, diastolic blood pressure; BMI, body mass index; FPG, fasting plasma glucose; HDL-C, high-density lipoprotein cholesterol; hs-CRP, high-sensitivity C-reactive protein; LDL-C, low-density lipoprotein cholesterol; TG, triglyceride; eGFR, estimated glomerular filtration rate; ACR, albumin to creatinine ratio; * *p* < 0.05; ** *p* < 0.01; *** *p* < 0.001.

**Table 3:** Associations between tertiles of hs-CRP and renal impairment.

Tertiles of hs-CRP	Model 1			Model 2				Model 3		
	OR	(95% CI)	*p*-Value	OR	(95% CI)		*p*-Value	OR	(95% CI)	*p*-Value
Tertiles of hs-CRP									
Low	1	–	–	1	–	–	1	–		–
Middle	1.49	(0.77–2.91)	0.24	1.52	(0.78–2.98)	0.22	1.42	(0.70–2.90)		0.33
High	2.25	(1.19–4.26)	0.01 *	2.22	(1.17–4.23)	0.02 *	2.16	(1.07–4.93)		0.03 *
*p*-Value for trend			0.01 *			0.02 *				0.03 *

Model 1: Unadjusted. Model 2: Multiple logistic regression adjusted for age and sex. Model 3: Multiple logistic regression adjusted for factors in model 2 plus smoking, BMI, SBP, LDL, FPG, Chinese herb use, and NSAID use. Abbreviations: hs-CRP, high-sensitivity C-reactive protein; RI, renal impairment; SBP, systolic blood pressure; BMI, body mass index; FPG, fasting plasma glucose; LDL-C, low-density lipoprotein cholesterol; OR, odds ratio; CI, confidence interval; * *p* < 0.05.

**Table 4:** Predictive values of hs-CRP for renal impairment in the total, middle-aged, and elderly groups.

Variables	AUC (95% CI)	*p*-Value	Cutoff Point	Sensitivity	Specificity
Total study population (*n* = 381)					
hs-CRP	0.60 (0.53–0.67)	0.01 *	1.61 mg/dL	0.56	0.66
Middle-aged group (<65 years, *n* = 219)					
hs-CRP	0.59 (0.49–0.69)	0.09	1.61 mg/dL	0.58	0.63
Elderly group (≥65 years, *n* = 162)					
hs-CRP	0.62 (0.51–0.72)	0.03 *	2.03 mg/dL	0.46	0.82

Abbreviations: hs-CRP, high-sensitivity C-reactive protein; AUC, area under the receiver operating characteristic curve; * *p* < 0.05.
